# Risk Factors and Pathogens of Wound Infection in Burn Inpatients from East China

**DOI:** 10.3390/antibiotics12091432

**Published:** 2023-09-11

**Authors:** Siqi Zhou, Shuzhen Xiao, Xuedong Wang, Xuefeng Wang, Lizhong Han

**Affiliations:** 1 Department of Laboratory Medicine, Ruijin Hospital, Shanghai Jiao Tong University School of Medicine, Shanghai 200025, China; zsq01g64@rjh.com.cn (S.Z.); xsz40689@rjh.com.cn (S.X.);; 2 Department of Clinical Microbiology, Ruijin Hospital, Shanghai Jiao Tong University School of Medicine, Shanghai 200025, China

**Keywords:** burn wound, burn wound infection, epidemiology, risk factors, resistance, pathogenesis

## Abstract

Background: Infection is the predominant contributor to morbidity and mortality in burn patients, and burn wound infection (BWI) is the most common reason. The objective of this research was to analyze the incidence, factors and progression of BWI, in terms of events and bacteria. Methods: Clinical variables of all qualified patients admitted to burn wards were analyzed retrospectively in 2021 at a tertiary hospital in eastern China through univariate analysis and multivariate logistic regression. The Kaplan–Meier method was also used for plotting survival curves. Isolates and resistance data were evaluated to demonstrate the evolution of targeted antibiotics of strains from BWI. Results: A total of 580 (median age, 39.5 years (23–56 years); 372/580 (64.14%) male) patients were evaluated, 348 (60.0%) of whom experienced BWI. A variety of factors are associated with BWI. Multivariate logistic regression analysis showed that depth and area of burn and duration from burn to first hospitalization are independent risk factors for BWI. For BWI onset in these patients, 47.24% (274/580) occurred in the first week. The most frequently isolated causative organism was *Staphylococcus aureus* (15.7%) in patients with BWI. The duration of transition from Gram-positive strains (median 3 days, (2–7 days)) to Gram-negative (median 10 days, (4–17 days)) ones isolated from burn wound shrunk. Hospital length of stay was considered as a protective factor for BWI. Conclusion: The precise assessment of factors affecting BWI in burn patients enhances prompt and suitable management. Swab cultures for surveillance could be utilized to monitor the microbiological status of burn patients.

## 1. Introduction

Burn injury ranked as the sixth leading cause of death in the 2013 update of the Global Burden of Diseases (GBD) [1]. Global hospitalizations for burn injuries reached 2.9 million in 2013, with 31 million individuals simultaneously requiring outpatient care. These alarming statistics also revealed a staggering death toll of 238,000 annually [1]. Age-standardized rates of incidence, mortality and DALYs (disability-adjusted life years) for burn injuries in 2017 were 108 new cases per 100,000, 0.7 deaths per 100,000, and 48 DALYs per 100,000 in China [2]. Research indicates that patients with burn-related infections have a mortality rate that is more than double that of patients without infections [3]. It was found that over 60% of burn victims died of infectious complications through a study supported by autopsy findings. Additionally, almost all of the patients had experienced at least one relevant organ failure [4]. Burn wound infection (BWI) is a significant contributor to sepsis and septic shock in severely ill individuals with burn injuries. Although, currently, more burn patients die of pneumonia than of burn wound infection, BWI remains a serious complication unique to burn recipients, and burn wound sepsis remains an important infectious complication in this population [5]. Burn wound colonization follows a certain timeline [6]. Following burn injuries, there may be significant increases in certain responses such as inflammatory cytokines, chemokines and metabolic hormones, which may vary depending on the time frame [7]. Managing the microflora within the burn wound is a constant challenge for clinicians, as it is essential to prevent potentially fatal complications through effective evaluation and treatment. Surface swab culture is a traditional, convenient and effective method, sufficient for the routine collection of multiple superficial wound samples used for the monitoring of organisms from burn wounds [8]. This article describes the infection process by evaluating the incidences, factors and evolution of wound infections, from the perspective of both events and bacteria, in order to achieve a deeper comprehension of BWI and analyze the characteristics of nosocomial infections to improve clinical care and preventive medication.

## 2. Results

### 2.1. Patient Demographics and Clinical Details

After excluding unrelated cases, a total of 580 qualified subjects were sifted from 736 patients admitted to the burn wards in 2021. The clinical characteristics of patients included are shown in Table 1. Their ages ranged from 0.5 to 93 years old (median 39.5 years, (IQR) 23–56 years), and 20.7% were 60 years old or older. Males were more numerous, accounting for 64.1%. The two wards can accommodate about 30 people each. The incidences of BWI in the two wards were similar. The percentage of total body surface area (% TBSA) ranged from 0.1% to 99%. Patients with burns that covered ≥ 20% TBSA comprised nearly 20% of the total burn patients. Patients with burns that covered more than 50% TBSA experienced an incidence of BWI that was up to 97.3%.

### 2.2. Burn Injury Event

#### 2.2.1. Related Incidence and Risk Factors

**Figure 1:**
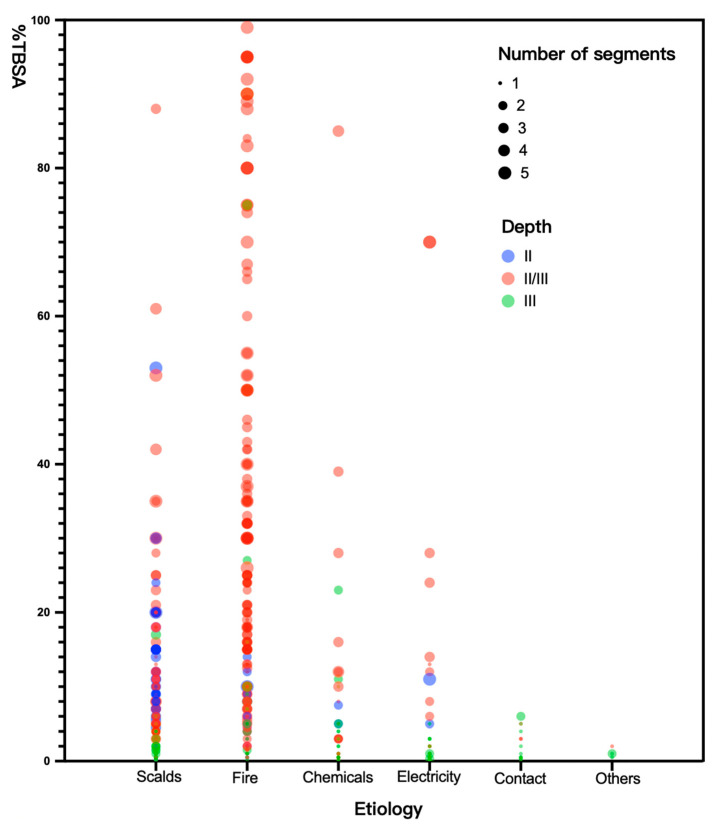
The association among etiology, depth, segments and % TBSA (the percentage of total body surface area) of burn. The X-axis of the bubble plot refers to the different etiologies of burn injuries, while the Y-axis refers to the TBSA of burns for each individual. The size of the bubbles indicates the number of affected anatomical segments for each patient, and the color represents the depth of the burn injuries. Depth II—burns extend into the underlying skin layer (dermis). Depth III—burns extend into full thickness. Depth II/III—Both of Depth II and III.

**Figure 2:**
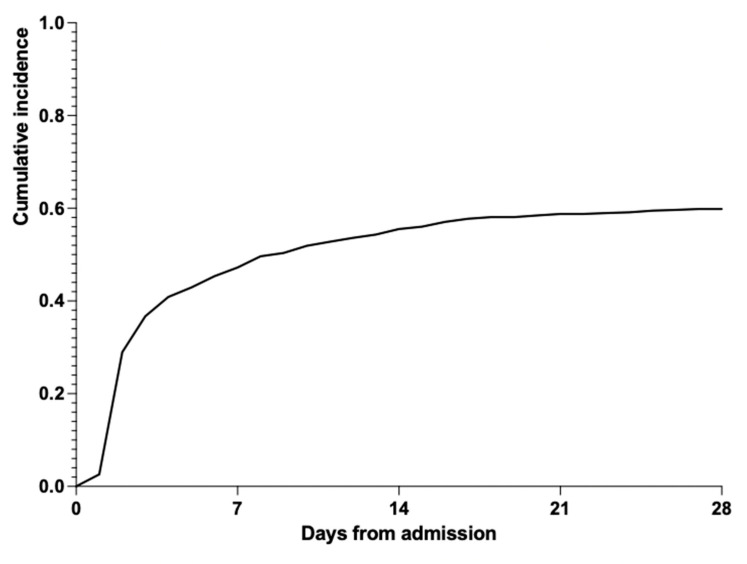
The cumulative incidence of infection from hospital admission to day 28.

Out of the 580 patients observed, 348 (348/580, 60%) were diagnosed with BWI, 135 (135/348, 38.8%) of whom had nosocomial infections. Among 4151 wound swab specimens, positive cultures accounted for 49.2% (2042/4151). The incidences of the two burn wards showed no significant difference, with a *p*-value of 0.759, indicating that there was no significant distinction in the microorganism environment between the two burn wards. The depth of burn wounds of the majority of patients (88.6%) reached grade III. More than half of patients presented with multiple burned anatomical segments. The majority had less than 20% TBSA burns in adults and less than 10% TBSA burns in children. Scalds made up the largest proportion, while electrical and fire injuries caused higher incidences of infection, suggesting that burn infection is apparently not a result of one factor. Figure 1 shows the relationship of the four factors of a burn event, displaying that fire injuries tend to cause wider, deeper and more severe burns. Despite a higher proportion of subjects, the degree of scalds was generally milder, and the areas were smaller. Patients who underwent three or more surgeries were at 1.5 times higher risk of BWI compared to others. Nearly half of patients who received medical treatment more than 3 days after injury (who tend to suffer a minor injury) still had the possibility of developing BWI. In total, 42.2% of patients with BWI who sought timely medical care (in 3 h) were diagnosed with nosocomial infections. The 28-day cumulative incidence is presented in Figure 2.

Univariate analysis showed that gender, depth, anatomical segments, %TBSA, inhalation injury, hypovolemia, etc., were associated with BWI (Table 1). The results of the multivariate logistic regression analysis showed that depth and % TBSA of burn and duration from burn to first hospitalization are independent risk factors for BWI (Table 1).

#### 2.2.2. Time of Appearance of First Infection 

**Figure 3:**
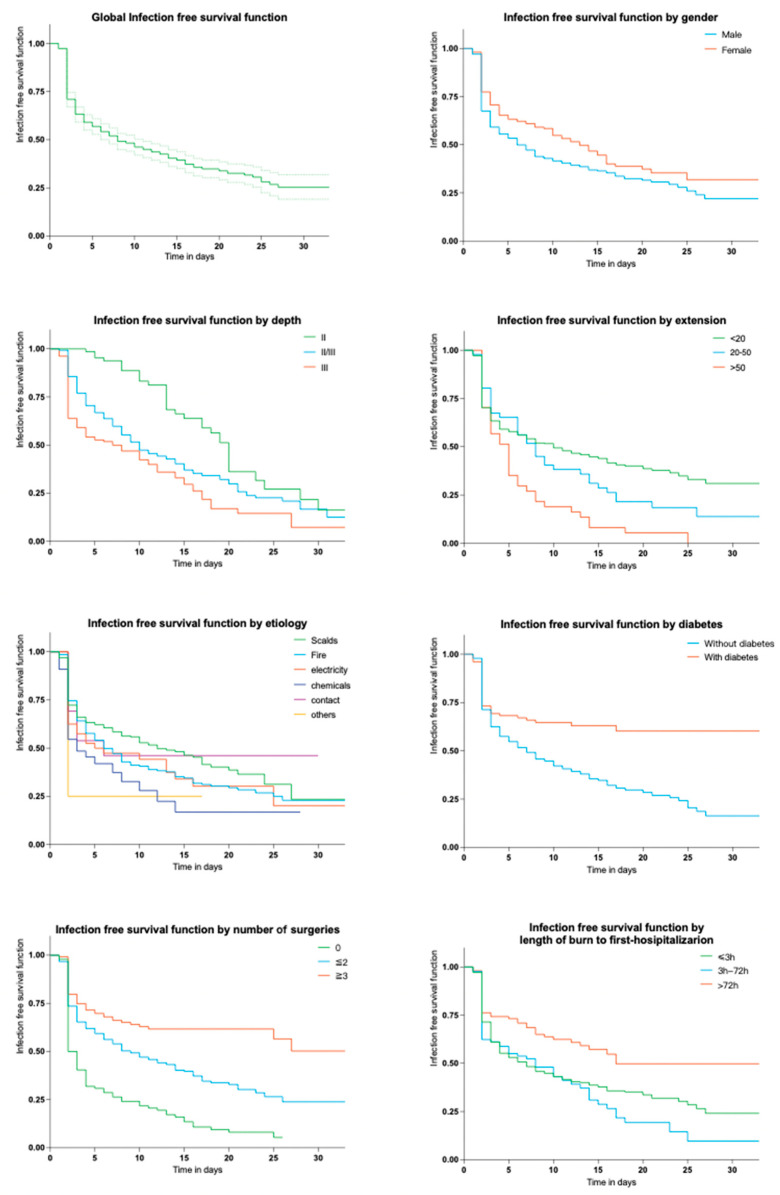
Kaplan–Meier curves of 30-days infection-free survival function by different factors. The dotted lines in the first subfigure mean 95% confidence interval of the global infection free survival function.

Global infection-free survival function was 19.0% at 62 days. A comparison was made to present the infection-free survival function under various factors (Supplementary Materials Table S1). Overall, it only took about one week for half of inpatients to develop wound infections. Interestingly, men tended to exhibit a faster rate of infection onset. The median time for 50% of scalds to develop infections was found to be only 2 days (Supplementary Materials Table S1), which may be attributed to the large sample size used for this investigation. As the depth and area of the burn increased, the median time was significantly shortened. This can also be seen in the Kaplan–Meier graphs in Figure 3.

### 2.3. Microorganisms

#### 2.3.1. Related Incidence and Antibiotic Resistance

Cultures of BWI swab specimens revealed a slight predominance of Gram-positive bacteria over Gram-negative bacteria (Table 2). Among Gram-positive bacteria, *Staphylococcus aureus* accounted for the largest proportion, followed by *Staphylococcus epidermidis*, *Enterococcus faecalis*, and *Staphylococcus hemolyticus*. Among Gram-negative bacteria, *Klebsiella pneumoniae* and *Acinetobacter baumanii* ranked first, followed by *Pseudomonas aeruginosa*. The resistance results indicated that *K. pneumoniae* was generally highly resistant to most antimicrobials (Table 3). *A. baumanii* was generally resistant to antimicrobials other than tetracyclines, colistin, and cefoperazone-sulbactam. Most Gram-negative isolates obtained were found to be multidrug-resistant. There are more drug options available for *Escherichia coli*. Overall, Gram-negative resistance was severe. All Gram-positive strains were susceptible to vancomycin (Table 4).

#### 2.3.2. Time of Appearance of Infection 

**Figure 4:**
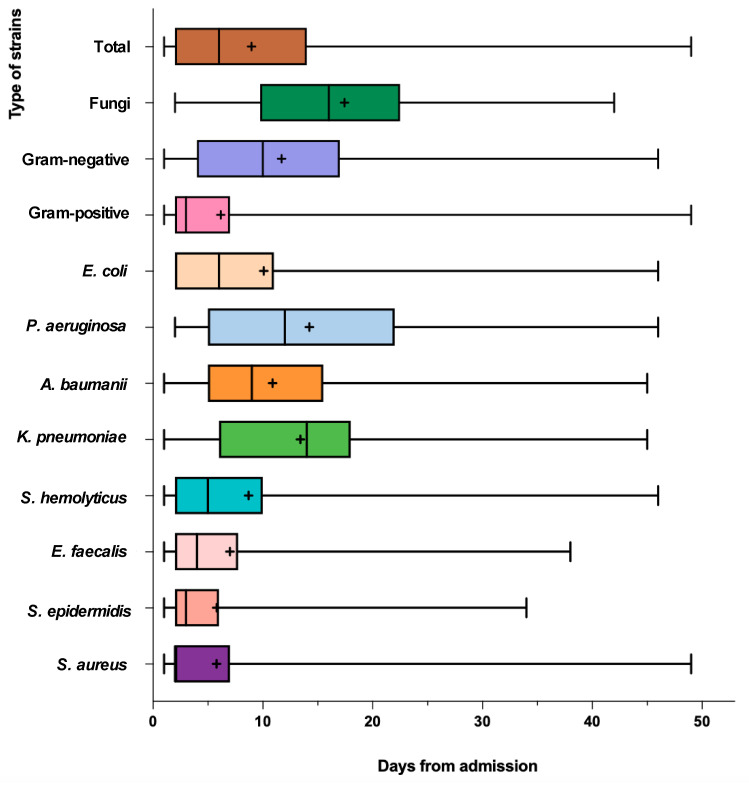
Boxplot representation of the time from injury to positive cultures by classes of microorganisms. Different strains are represented by different colors. ‘+’ —the mean. The vertical line—the median.

The times taken for various strains to appear culture-positive for the first time were recorded (Table 2). *S. aureus* poses an infection risk within 2 days of hospital admission (median 2 days, (IQR) 2–7 days),whereas exposure to *P. aeruginosa* carries the longest risk period (median 12 days, (IQR) 5–22 days). *K. pneumoniae* has the latest positive time. Generally, the threat of *Staphylococcus* infection peaks approximately in the first week after hospitalization. Figure 4 also illustrates the results.

## 3. Discussion

The aim of this study was to clarify potential factors of BWI and describe the epidemiology of surface swab cultures in burn patients, revealing the characteristics of burn injury events and microorganisms of BWI, including incidences, evolution and factors. It is not common to collect swab specimens to demonstrate the development of dominant bacteria of BWI. Furthermore, BWI is known to advance to invasive infection and sepsis. This makes early detection, diagnosis and treatment critical to the overall outcome for the patient.

The global incidence of BWI was recorded at 60%, which is comparatively similar to some of preceding investigations in the UK (68%) [9] and Morocco (68.2%) [10]. Additionally, the collective incidence of positive swabs is 49.2% (2042/4151), which is also lower when compared to a prior study [11]. In the present study, male subjects were nearly twice as likely as female patients to develop BWI (64.1% vs. 35.9%), similar to the aforementioned studies [11]. The median age was 39.5 (23–56) years, which correlates with the findings reported by other studies [10,12]. Our data show that many variables are influencing factors of BWI, for example, gender, depth, area, length of burn to first hospitalization, etc. The results are consistent with other studies [3,13,14,15]. Scalds were the most frequent cause, accounting for nearly half of the cases, followed by fire, as reported by Jaimes S L et al. [15]. Interestingly, the incidence of scalds did not rank highest among the observed injuries; however, it exhibited a swift progression, which is potentially associated with the extensive size of the sample population itself. 

Interestingly, our study highlights that the duration of hospital stay plays a crucial role in minimizing the risk of BWI: previous research found that the possibility of BWI can be significantly decreased if the incision is made within 24–48 h of injury [16]. This early surgical intervention may reduce blood loss, the occurrence of infections, hospital stay and mortality rate and increase the effectiveness of grafting. We reported an incidence of BWI of 2.6% on day 1 after admission (Figure 1), and it rose to 29.0% on day 2; then, it became stable during the following days, supporting the fact that hospital length of stay is inutile for the development of infections. Additionally, removing dead tissue promptly can limit the production of chemical mediators and help alleviate inflammation and multisystem organ failure, which may be one of the reasons why the cumulative incidence leveled out two days after admission. At the same time, skin infections occur earlier during hospitalization, generally within the first week, conforming with our results [17].

In our study, *S. aureus* was the most common bacterial organism colonizing burn wounds, accounting for 15.7% of all isolates. Similar findings have been reported in other epidemiological studies of burn wound infections [15], while some other studies report *P. aeruginosa* as the predominant organism [18,19]. In fact, both of them are common pathogens detected in BWI [11]. Carbapenems showed surprisingly high resistance levels among most Gram-negative rods (basically above 70%). All Gram-positive strains were susceptible to vancomycin. The prevalence rate of MRSA (51.0%) was similar to another result obtained in our center from 2011 to 2016 [20]. Related findings confirm that infections via Gram-positive organisms are characteristic of the early period of burn injuries. Subsequently, Gram-negative organisms gradually start to take over as the wounds get colonized with microbes originating either from the patient’s respiratory and gastrointestinal flora or transferred through healthcare workers or the hospital environment. Then, with delayed wound closure and an increase in the need for broad-spectrum antibiotics, further replacement with fungi and antibiotic-resistant bacteria takes place [5]. Our research found that BWI was identified within 1 to 96 days after hospitalization (median 6 days, (IQR) 2–14 days). A study conducted at a single center on 5524 patients with burn injuries admitted between 2004 and 2013 [21] demonstrated that the median time from admission to first positive culture was 3 days ((IQR) 2–8 days) for S. aureus vs. 18 days ((IQR) 9–36 days) for P. aeruginosa. In contrast to previous research endeavors, it has been observed that although the trend of transitioning from Gram-positive organisms to Gram-negative ones remains unchanged, the duration of this transition has been reduced. Thus, it is crucial to manage infection during the first week after the hospitalization of burn patients and prevent the early emergence of Gram-negative pathogens in time, which includes a variety of antibiotic-resistant bacteria. Therefore, removing the eschar and promptly dressing the wound is essential for preventing infection in burn patients, and complete debridement should be performed within a maximum of two weeks at our burn center. 

This study has several limitations. Firstly, it is a retrospective monocentric study, with a limited number of subjects and observed events. Despite the fact that our data were only gathered for one single year, the sample size is significant. As the study was conducted retrospectively, clinical data were occasionally incomplete. Therefore, we depended on the final diagnosis provided by the clinician. Furthermore, the microbial cultures obtained from skin lesions were classified as instances of skin colonization. It is possible that the microorganisms isolated from these cultures were simply colonizers and not necessarily responsible for causing an infection. However, these bacteria make up a small proportion in our study. *Coagulase-negative staphylococcus, S. aureus, P. aeruginosa, K. pneumoniae* and *A. baumanii* are usually considered non-colonizers.

## 4. Materials and Methods

### 4.1. Study Design and Setting

We conducted a retrospective cohort study at a tertiary hospital between 1 January 2021, and 31 December 2021. The burn department in Ruijin Hospital is situated in Shanghai, East China, which is the largest burn center in the eastern region of China and frequently receives patients with large % TBSA (total body surface area) burns referred from other centers. There are a total of 100,000 outpatient and emergency visits annually. The total number of discharged patients reaches 900 each year in Ruijin Hospital. Patients who were hospitalized for the first time in the burn ward and sent for cultures of swab specimens were included in our study.

The following exclusion criteria were established:

1. Patients for non-burn reasons such as scar plastic surgery or chronic persistent infections;
2. Patients who had been taking immunosuppressive drugs such as glucocorticoids for a long time or had serious autoimmune diseases;
3. Patients who were uncooperative due to mental disorders or special circumstances and insisted on being discharged against medical advice or treatment voluntarily;
4. Patients with inadequate clinical data.

Data were extracted from medical records via the electronic hospital and laboratory information system, including demographic characteristics, etiology, area, depth and anatomical segments of burn, inhalation injury, hypovolemia, diabetes, duration from burn to first hospitalization, hospital length of stay, date of first positive scrab culture, identification of isolates, antimicrobial resistance condition, surgeries for burn wounds and outcomes.

### 4.2. Definitions

The definition of BWI followed the relevant guidelines (Supplementary Materials Table S2) [22]. The ‘rule of nines’ was used to assess % TBSA [23] by senior burn clinicians after admission. Our study categorizes burn depth into three degrees. First-degree means burns extending to superficial thickness, affecting the epidermis only.Burns extending into the underlying skin layer (dermis) are classed as second-degree. Third-degree means full thickness [24]. ‘Surgery’ in our study was defined as surgery for burn wounds. Nosocomial infections were defined as positive samples taken more than 48 h after hospital admission with no evidence of any clinical signs or symptoms of infection between hospital admission and the onset of infection. For this survival analysis, the time of admission was designated as the time of origin, and survivors were censored at the time of discharge. The time of first positive cultures was defined as the deadline for BWI patients. The time of discharge was considered as the deadline for patients that did not develop BWI. This article set the survival rate at 30 days since wound infections usually occur sooner. The global cumulative incidence was established to track the progression of BWIs over time.

### 4.3. Microbiology

Certain patients may experience recurring instances of contracting the same organism; hence, this study primarily focused on any distinctive isolations or initial growths of an organism within an individual patient. Duplicate strains found from the same patient and location were excluded, and the identified initial strains and distinct strains found from the same specimen were retained. All strains were isolated from wound secretions and exudate samples of inpatients admitted to the burn ward of Ruijin Hospital from 1 January 2021 to 31 December 2021. The identification of strains isolated was performed using MALDITOF MS (bioMérieux, Marcy l’Etoile, France). An antimicrobial susceptibility test was performed using VITEK®2 Compact (bioMérieux, Marcy l’Etoile, France). The results of susceptibility experiments were interpreted according to the 2021 Clinical and Laboratory Standard Institute (CLSI) standard [25]. The susceptibility paper sheets were purchased from Oxoid. *Escherichia coli* ATCC 25922, *Escherichia coli* ATCC35218, *Staphylococcus aureus* ATCC25923, *Staphylococcus aureus* ATCC25213 and *Pseudomonas aeruginosa* ATCC27853 were used as quality control strains.

### 4.4. Statistical Analysis

SPSS version 26.0 (accessed on 9 December 2022, IBM, Armonk, NY, USA) and GraphPad Prism version 9.0.0 (GraphPad Software, San Diego, CA, USA, www.graphpad.com.) were used to conduct statistical analyses and generate figures. Descriptive statistics were utilized to provide an overall understanding of the included patients’ characteristics. Categorical variables were presented through frequencies and percentages, while normality was assessed for continuous variables. Normally distributed variables were reported using the mean and standard deviation (SD), whereas non-normally distributed variables were displayed using the median and interquartile range (IQR). Categorical variables were compared using the chi-square test and Fisher’s exact test, while the Student T test or the Mann–Whitney U test was used to identify differences in continuous variables. Univariate analysis was used to assess potential risk factors for infection, and variables with a *p*-value < 0.05 were further analyzed using multivariate logistic regression to determine factors that were independently associated with BWI. In the meantime, odds ratios (OR) and 95% confidence intervals were calculated. We used the Kaplan–Meier method to plot survival curves and assessed differences by means of the log-rank test. All *p*-values were two-tailed, and *p* < 0.05 was considered statistically significant.

## 5. Conclusions

Bioinformatic and phylogenetic studies are warranted to better understand the phylogeny and pathogenicity of these isolates in the future. Furthermore, we discovered that the strains originating from BWI exhibit notable dissimilarities throughout the patient’s admission, as indicated by the results of swab cultures. These findings could aid in predicting the causative agents of BSI in burn patients [18]. Burn centers are advised to identify predominant microbial isolates at their facility and track changes in the microbiology of burn wounds, including antibiotic resistance. This facilitates the creation of efficient escalation therapies and protocols for first-line antimicrobial treatment. The establishment of antimicrobial stewardship initiatives and strict policies for infection control and surveillance would probably also enhance outcomes for patients in burn units.

## Figures and Tables

**Table 1:** Clinical characteristics of 580 qualified patients in burn wards and their relationships with infection.

Characteristic	Total (*n* = 580)	BWI (*n* = 348)	Incidence	Univariate		Multivariate	
				OR (95% CI)	*p*-Value	aOR (95% CI)	*p*-Value
Age(years, median, IQR)	39.5 (23–56)	41 (24–57)	60.0%	1.00 (1.00–1.01)	0.552		
Age ≥ 60	120 (20.7%)	71 (20.4%)	59.2%	0.99 (0.95–1.03)	0.631		
Ward							
I	318 (54.8%)	189 (54.3%)	59.4%	-	-		
II	262 (45.2%)	159 (45.7%)	60.7%	1.05 (0.75–1.47)	0.759		
Gender							
Male	372 (64.1%)	240 (69.0%)	64.5%	-	-		
Female	208 (35.9%)	108 (31.0%)	51.9%	1.68 (1.19–2.38)	0.003	0.68 (0.46–1.00)	0.048
Depth							
II	66 (11.4%)	35 (10.0%)	53.0%	-	-		
II/III	278 (47.9%)	184 (52.9%)	66.2%	0.94 (0.52–1.62)	0.814	2.39 (1.20–4.78)	0.014
III	236 (40.7%)	129 (37.1%)	54.7%	1.62 (1.14–2.32)	0.008	2.84 (1.43–5.67)	<0.001
Anatomical segments							
Limbs	535 (92.2%)	325 (93.4%)	60.8%	1.48 (0.81–2.72)	0.207		
Torso	217 (37.4%)	150 (43.1%)	69.1%	1.87 (1.31–2.66)	0.001		
Head and neck	246 (42.4%)	163 (46.8%)	66.3%	1.58 (1.12–2.23)	0.008		
Hip	70 (12.1%)	59 (17.0%)	84.3%	4.10 (2.11–7.99)	<0.001		
Perineum	53 (9.1%)	45 (12.9%)	84.9%	4.16 (1.92–9.00)	<0.001		
TBSA (%)							
<20	458 (79.0%)	247 (71.0%)	55.3%	-	-		
20–50	85 (14.7%)	65 (18.7%)	76.5%	3.32 (1.57–7.02)	0.002	7.41 (3.00–18.28)	<0.001
>50	37 (6.4%)	36 (10.3%)	97.3%	29.06 (3.95–18.77)	0.001	26.43 (7.88–49.48)	<0.001
Total	5 (1–16)	5 (1–16)	60.0%	1.05 (1.03–1.06)	<0.001		
Inhalation injury	149 (25.7%)	107 (30.8%)	71.8%	2.01 (1.34–3.01)	<0.001		
Hypovolemia	167 (28.8%)	128 (36.8%)	76.7%	2.88 (1.92–4.33)	<0.001		
Etiology							
Scalds	289 (49.8%)	149 (42.8%)	51.6%	-	-		
Fire	201 (34.7%)	138 (39.7%)	68.7%	2.06 (1.41–3.00)	<0.001		
Chemicals	40 (6.9%)	27 (7.8%)	67.5%	2.51 (1.13–5.58)	0.024		
Electricity	33 (5.7%)	24 (6.9%)	72.7%	1.95 (0.97–3.93)	0.61		
Contact	13 (2.2%)	7 (2.0%)	53.9%	1.10 (0.36–3.34)	0.872		
Others	4 (0.7%)	3 (0.9%)	75.0%	2.82 (0.29–27.42)	0.372		
Diabetes	101 (17.4%)	69 (19.8%)	68.3%	1.54 (0.97–2.43)	0.065		
Surgery							
0	94 (16.2%)	49 (14.1%)	52.1%	-	-		
1/2	363 (62.6%)	194 (55.8%)	53.4%	1.05 (0.67–1.66)	0.82	0.13 (0.06–0.28)	<0.001
≥3	123 (21.2%)	105 (30.2%)	85.4%	5.36 (2.82–10.19)	<0.001	0.04 (0.03–0.10)	<0.001
Total	2 (1–2)	2 (1–2)	60.0%	1.60 (1.38–1.85)	<0.001		
Duration from burn to first hospitalization (h)							
≤3 h	382 (65.9%)	250 (71.8%)	65.5%	-	-		
3 h–72 h	85 (14.7%)	42 (12.1%)	49.4%	1.86 (1.19–2.89)	0.006	4.21 (2.30–7.70)	<0.001
>72 h	101 (17.4%)	51 (14.7%)	50.5%	0.96 (0.54–1.71)	0.883	5.03 (2.30–11.02)	<0.001
Total	2 (2–12)	2 (2–4)	60.0%	1.00 (1.00–1.00)	0.368		
Hospital length of stay, days (median, IQR)	17(10–26)	15 (9.75–22)	60.0%	0.97(0.96–0.98)	<0.001	0.97(0.96–0.99)	0.002

BWI—burn wound infection. OR—odds ratio. CI—confidence interval. aOR—ajusted odds ratio. Ward I, Ward II—the number of two different burn wards of Ruijin Hospital. Depth II—burns extend into the underlying skin layer (dermis). Depth III—burns extend into full thickness. Depth II/III—Both of Depth II and III. ‘-’—control groups. TBSA(%)—the percentage of total burn surface area. ’h’—hour. IQR—interquartile range.

**Table 2:** Frequency of first isolated pathogens from burn wounds in burn patients and time between admission and first positive swab culture.

First Isolated Pathogen	Frequency (*N*/%)	Time Between Admission and First Positive Swab Culture
Gram-positive	343 (52.9%)	3 (2–7)
*S. aureus*	102 (15.7%)	2 (2–7)
MRSA	52 (8.0%)	2 (1–9)
MSSA	50 (7.7%)	1 (1–2)
*S. epidermidis*	92 (14.2%)	3 (2–6)
*Enterococcus faecalis*	44 (6.8%)	4 (2–7)
*S. hemolyticus*	38 (5.9%)	5 (2–10)
Others	67 (10.3%)	-
Gram-negative	269 (41.5%)	10 (4–17)
*K. pneumoniae*	69 (10.6%)	14 (6–18)
*A. baumanii*	69(10.6%)	9 (5–15.5)
*P. aeruginosa*	55 (8.5%)	12 (5–22)
*Escherichia coli*	13 (2.0%)	6 (2–11)
Others	63 (9.7%)	-
Fungi	37 (5.7%)	16 (9.75–22.5)
Total		6 (2–14)

*N*—the number of strains. MRSA—methicillin-resistant *Staphylococcus aureus*.

**Table 3:** Antibacterial resistance of common Gram-negative microorganisms in BWI patients.

Antibiotics	*K. pneumoniae* (*N* = 69)		*A. baumanii* (*N* = 69)		*P. aeruginosa* (*N* = 55)		*Escherichia coli* (*N* = 13)	
	*n*	Resistance (%)	*n*	Resistance (%)	*n*	Resistance (%)	*n*	Resistance (%)
Cefazolin	63	91.3%	69	100.0%	-	-	10	76.9%
Cefuroxime	64	92.8%	67	97.1%	-	-	8	61.5%
Ceftazidime	58	84.1%	65	94.2%	16	29.1%	4	30.8%
Ceftriaxone	62	89.9%	68	98.6%	-	-	8	61.5%
Cefepime	57	82.6%	64	92.8%	18	32.7%	4	30.8%
Aztreonam	58	84.1%	-	-	13	23.6%	2	15.4%
Imipenem	51	73.9%	65	94.2%	41	74.6%	1	7.7%
Meropenem	51	73.9%	65	94.2%	39	70.9%	2	15.4%
Ampicillin-sulbactam	60	87.0%	61	88.4%	-	-	4	30.8%
Piperacilin-tazobactam	54	78.3%	65	94.2%	19	34.6%	1	7.7%
Ticarcillin-clavulanate	55	79.7%	65	94.2%	39	70.9%	1	7.7%
Cefoperazone-sulbactam	55	79.7%	33	47.8%	38	69.1%	1	7.7%
Amikacin	50	72.5%	52	75.4%	37	67.3%	1	7.7%
Tobramycin	50	72.5%	59	85.5%	38	69.1%	3	23.1%
Ciprofloxacin	61	88.4%	65	94.2%	40	72.7%	8	61.5%
Levofloxacin	61	88.4%	61	88.4%	41	74.6%	7	53.9%
Doxycycline	57	82.6%	24	34.8%	-	-	7	53.9%
Minocycline	54	78.3%	3	4.4%	-	-	6	46.2%
Tigecycline	-	-	2	2.9%	-	-	0	0.0%
Colistin	11	16.0%	2	2.9%	2	3.6%	1	7.7%
Fosfomycin	53	76.8%	55	79.7%	-	-	3	23.1%
Trimethoprim-sulfamethoxazole	59	85.5%	66	95.7%	-	-	9	69.2%

*N*—the number of strains. *n*—the number of resistant strains.‘-’—data is not available.

**Table 4:** Antibacterial resistance of *S. aureus* in BWI patients.

Antibiotics	*S. aureus* (*N* = 102)		MRSA (*N* = 52)		MSSA (*N* = 50)	
	*n*	Resistance	*n*	Resistance	*n*	Resistance
Penicillin	94	92.2%	52	100.0%	42	84.0%
Oxacillin	53	52.0%	52	100.0%	1	2.0%
Gentamicin	13	12.8%	12	23.1%	1	2.0%
Ciprofloxacin	23	22.6%	15	28.9%	8	16.0%
Levofloxacin	24	23.5%	15	28.9%	9	18.0%
Moxifloxacin	22	21.6%	15	28.9%	7	14.0%
Clindamycin	40	39.2%	25	48.1%	15	30.0%
Erythromycin	45	44.1%	28	53.9%	17	100.0%
Rifampin	6	5.9%	6	11.5%	0	0.0%
Vancomycin	0	0.0%	0	0.0%	0	0.0%
Linezolid	0	0.0%	0	0.0%	0	0.0%
Trimethoprim-sulfamethoxazole	13	12.8%	10	19.2%	3	6.0%
Quinupristin-dalfopristin	1	1.0%	1	1.9%	0	0.0%
Tetracycline	25	24.51%	21	40.4%	4	8.0%

*N*—the number of strains. *n*—the number of resistant strains. MRSA—methicillin-resistant *Staphylococcus aureus*. MSSA—methicillin- sensitive *Staphylococcus aureus*.

## Data Availability

The datasets generated and/or analyzed during the current study are not publicly available but are available from the principal investigator (PI) on reasonable request.

## Supplementary Materials

The following supporting information can be downloaded at https://www.mdpi.com/article/10.3390/antibiotics12091432/s1, Table S1. Clinical characteristics of 580 patients in burn wards and their relationship with infection and survival functions, Table S2. Criteria for diagnosis of burn wound infections [26].
